# Are long‐term widespread avian body size changes related to food availability? A test using contemporaneous changes in carotenoid‐based color

**DOI:** 10.1002/ece3.2739

**Published:** 2017-03-31

**Authors:** Roellen Little, Janet L. Gardner, Tatsuya Amano, Kaspar Delhey, Anne Peters

**Affiliations:** ^1^ School of Biological SciencesMonash UniversityClaytonVic.Australia; ^2^ Division of Evolution, Ecology and GeneticsThe Australian National UniversityCanberraACTAustralia; ^3^ Conservation Science GroupDepartment of ZoologyUniversity of CambridgeCambridgeUK

**Keywords:** Bergmann's rule, carotenoids, global change, plumage reflectance

## Abstract

Recent changes in global climate have been linked with changes in animal body size. While declines in body size are commonly explained as an adaptive thermoregulatory response to climate warming, many species do not decline in size, and alternative explanations for size change exist. One possibility is that temporal changes in animal body size are driven by changes in environmental productivity and food availability. This hypothesis is difficult to test due to the lack of suitable estimates that go back in time. Here, we use an alternative, indirect, approach and assess whether continent‐wide changes over the previous 100 years in body size in 15 species of Australian birds are associated with changes in their yellow carotenoid‐based plumage coloration. This type of coloration is strongly affected by food availability because birds cannot synthesize carotenoids and need to ingest them, and because color expression depends on general body condition. We found significant continent‐wide intraspecific temporal changes in body size (wing length) and yellow carotenoid‐based color (plumage reflectance) for half the species. Direction and magnitude of changes were highly variable among species. Meta‐analysis indicated that neither body size nor yellow plumage color showed a consistent temporal trend and that changes in color were not correlated with changes in size over the past 100 years. We conclude that our data provide no evidence that broad‐scale variation in food availability is a general explanation for continent‐wide changes in body size in this group of species. The interspecific variability in temporal changes in size as well as color suggests that it might be unlikely that a single factor drives these changes, and more detailed studies of museum specimens and long‐term field studies are required to disentangle the processes involved.

## 1 Introduction

Recent anthropogenic‐induced changes in climate have led to a broad range of biological responses, including changes in the timing of major life events such as breeding and migration and shifts in species’ distributions (Walther et al., 2002). More recently, morphological changes have been recognized as a pervasive response to recent climate change, in particular widespread changes in animal body size (Daufresne, Lengfellner, & Sommer, 2009; Gardner, Peters, Kearney, Joseph, & Heinsohn, 2011; Sheridan & Bickford, 2011; Yom‐Tov, Benjamini, & Kark, 2002; Yom‐Tov & Geffen, 2011). Recent studies demonstrate that declining body size appears to be more common than increasing body size, particularly in the case of birds (Gardner, Amano, Backwell, et al., 2014; Sheridan & Bickford, 2011; Teplitsky & Millien, 2014; Van Buskirk, Mulvihill, & Leberman, 2010; Yom‐Tov & Geffen, 2011). However, while body size declines may be frequent, they are certainly not universal: even closely related species from the same bioregion may show increases, decreases, and no change in size (e.g., Gardner, Heinsohn, & Joseph, 2009; Gardner, Amano, Backwell, et al., 2014) and patterns of change within species may vary for different morphological size traits (Salewski, Siebenrock, Hochachka, Woog, & Fiedler, 2014).

Several mechanisms could contribute to size changes as a correlate of contemporary climate change in a global context. Recent changes in body size are often interpreted in the context of Bergmann's Rule, originally proposed to explain geographic variation in body size (Bergmann, 1847). The original explanation for the rule involved surface area to volume ratios and heat conservation mechanisms in endotherms and predicts a mean increase in body size with latitude as an adaptive thermoregulatory response to colder climates (Bergmann, 1847; Scholander, 1956). Thus, recent temporal declines in body size may be the result of selective advantages of smaller body size that allow more efficient heat dissipation in warmer climates (Gardner et al., 2011; but see Teplitsky & Millien, 2014). However, contemporary climate change involves a number of additional processes that could affect body size, and contribute to the observed variation in size responses.

Climate‐driven changes in primary productivity or habitat quality that affect an animal's resource availability (net energy balance) could equally account for observed global size change patterns (McNab, 2010). For example, Huston and Wolverton (2011) propose that ecologically relevant primary productivity (eNPP) during the growing season may underlie latitudinal size patterns described by Bergmann's Rule. Indeed, many studies suggest that recent changes in body size may result from climate‐driven changes in primary productivity that affect food availability or food quality (Gienapp, Teplitsky, Alho, Mills, & Merilä, 2008; Goodman et al., 2012; Millien et al., 2006; Ozgul et al., 2010; Teplitsky, Mills, Alho, Yarrall, & Merilä, 2008; Yom‐Tov & Geffen, 2011). In particular, changing food availability may help to explain observed variation in contemporary body size changes: changes in primary productivity will vary across landscapes due to the considerable variation in rainfall patterns that strongly determine primary production (Rosenzweig, 1968). Testing this hypothesis directly over long time periods, however, proves difficult as we lack long‐term records of primary productivity (Roxburgh et al., 2004). An alternative approach is to assess whether temporal changes in size are matched with temporal changes in other phenotypic traits that are tightly linked with food availability and that are preserved in historical specimens (Gardner et al., 2011), which is the approach we follow here.

In this study, we use carotenoid pigmentation of plumage as a proxy that could provide independent evidence for changes in food availability over the last 100 years. Carotenoids are plant pigments responsible for most yellow, orange and red colors of animals, including birds (McGraw, 2006). To obtain carotenoid pigments, animals must ingest them either directly from an herbivorous diet, or indirectly via the food chain (reviewed in McGraw, 2006). As pigments are obtained via the diet, natural fluctuations or environmental modification have significant impacts on plumage color (Ewen et al., 2006; Isaksson 2009; Linville & Breitwisch, 1997; Slagsvold & Lifjeld, 1985). For example, vegetation structure can explain variation in plumage colors of insectivorous songbird nestlings (Arriero & Fargallo, 2006; Slagsvold & Lifjeld, 1985), while extreme weather causing low fruit availability resulted in reduced plumage colors in an omnivorous songbird (Linville & Breitwisch, 1997). Expression of plumage colors is linked not only with access to pigments, but also with general nutritional condition of birds at the time of molt (Chui et al. 2011; Hill & Montgomerie, 1994; Hill, 2000; Jacot & Kempenaers, 2007; McGraw, Hill, & Parker, 2005; Peters, Delhey, Johnsen, & Kempenaers, 2006; Peters, Delhey, Andersson, van Noordwijk, & Förschler, 2008; Peters et al., 2011; Shawkey, Hill, McGraw, Hood, & Huggins, 2006) and could also reflect food availability this way. Because carotenoids deposited in feathers are chemically inert, carotenoid‐based plumage color of historical specimens is an accurate representation of color of live birds (Doucet & Hill, 2009). Thus, the link between carotenoid‐based plumage color, dietary intake, and nutritional condition provides a historical window on recent changes in food availability.

Here, we measure body size and plumage color from museum specimens of 15 bird species, sampled across their geographic range over the past 100 years, in order to detect species‐level patterns of change. Our study aims to determine whether temporal changes in size and carotenoid‐based plumage colors of Australian birds are correlated within species, consistent with the idea that wide‐ranging changes in food availability could be driving large‐scale morphological changes over time.

## 2 Materials and Methods

### 2.1 Study species and specimens

We selected 15 Australian bird species from three families in the Meliphagoidea superfamily (Table 1) that have similar ecology (mostly/partly insectivorous and nectarivorous), and an unambiguous carotenoid‐based plumage patch (for details see Table S1). Previous work demonstrated considerable variation in temporal patterns of body size change among species within the superfamily (Gardner, Amano, Backwell, et al., 2014; Gardner, Amano, Mackey, et al., 2014). As we hypothesize that the direction of color change should correlate positively with the direction of body size change, variation in the patterns of body size change should maximize the power to detect such an effect.

**Table 1:** Summary of linear mixed effect models assessing temporal change in body size (wing length, mm) and carotenoid‐based plumage color (reflectance PC1, jnd) across 15 species of Australian passerine birds. For details of species and specimens see Table S1; full model outputs for each species are in Table S2 and S3. EDF = Effective degrees of freedom based on GAMMs, where values above 3 indicate nonlinear patterns of temporal change (see ‘Materials and Methods’ for more details); β = slope of the linear temporal effect: mm/year, jnd/year). Models that indicate significant (*p* ≤ .05) temporal changes are highlighted in bold

Species	Wing length						Carotenoid‐based plumage color					
	EDF	β	*SE*	*df*	*t*	*p*	EDF	β	*SE*	*df*	*t*	*p*
Buff‐rumped thornbill	1	−.00613	.006	107	−0.29	.771	2.86	−.00154	.004	103	−0.35	.726
Yellow thornbill	1	−.00037	.005	96	−0.08	.940	1	−.00172	.005	94	−0.35	.725
Yellow‐rumped thornbill	0.99	−.00212	.004	130	−0.49	.626	1	.00772	.005	126	1.57	.119
Weebill	1	.00282	.003	153	0.97	.336	**1**	−**.01089**	**.003**	**145**	−**3.52**	**.001**
White‐throated gerygone	**3.81**	−**.03041**	**.005**	**75**	−**5.67**	**<.001**	4.41	−.00732	.005	71	−1.39	.168
Spotted pardalote	1	−.00423	.004	137	−1.11	.270	**1**	**.01844**	**.009**	**130**	**2.06**	**.042**
Yellow‐tinted honeyeater	1	−.00225	.011	59	−0.21	.833	**1**	−**.03524**	**.010**	**57**	−**3.66**	**.001**
White‐plumed honeyeater	**1.69**	−**.01949**	**.007**	**133**	−**2.89**	**.004**	**1.53**	−**.02224**	**.004**	**130**	−**5.18**	**<.001**
Singing honeyeater	1.8	.00478	.007	110	0.64	.521	1	.00519	.005	103	1.02	.308
Yellow‐throated miner	1	−.01499	.011	130	−1.31	.193	1	.00315	.004	128	0.86	.392
Yellow‐tufted honeyeater	1	−.00728	.011	124	−0.63	.527	1.98	.00964	.008	121	1.28	.204
Lewin's honeyeater	1	.00167	.008	92	0.21	.831	1	−.00489	.004	88	−1.26	.211
New Holland honeyeater	1	−.00728	.008	134	−0.86	.392	**1**	**.01083**	**.005**	**127**	**1.98**	**.050**
White‐naped honeyeater	2.22	−.01110	.008	103	−1.39	.169	**1**	**.01530**	**.005**	**98**	**2.91**	**.004**
White‐eared honeyeater	**1**	−**.02009**	**.007**	**113**	−**2.75**	**.007**	2.89	.00024	.005	108	0.05	.962

In total, 1,804 specimens (65–162 per species) collected between 1900 and 2008 were measured from the National Museum of Victoria, Melbourne and the Australian National Wildlife Collection, Canberra. This time frame encompasses both pre and postwarming conditions in Australia (CSIRO and Bureau of Meteorology 2007, http://www.climatechangeinaustralia.gov.au/technical_report.php), therefore providing us with baseline conditions prior to recent climate change (Lister & Climate Change Research Group, 2011). We sampled individuals across the geographic range of each species (see Fig. S1 for maps depicting sampling localities) to minimize effects of local factors that can affect body size (e.g., interspecific competition, predation pressure). Our aim was to detect species‐level response to broad‐scale environmental change, in order to test predictions of overarching mechanisms.

For each individual measured, the registration number, year of collection, and sex were recorded from museum tags. Location of collection (latitude and longitude) was obtained from meta‐data provided by Online Zoological Collections of Australian Museums (OZCAM, http://ozcam.org.au/). Altitude was calculated using ArcGIS, using a digital elevation map from Geoscience Australia (http://www.ga.gov.au/home). Subspecies were determined by collection location and distribution maps in Schodde and Mason (1999). Season of collection was assigned using the month of collection provided by OZCAM. No juveniles, birds in molt, highly damaged or unsexed individuals were measured.

### 2.2 Body size measurement

Wing length is considered to be the best single linear predictor of structural body size and was used as an index of body size in this study (Gosler, Greenwood, Baker, & Davidson, 1998). Some authors have used tarsus or culmen length as an index of body size, because these traits are less variable across an individual's life. However, both are subject to Allen's rule, which predicts a decrease in such traits with increasing latitude, opposite to Bergmann's rule (Symonds & Tattersall, 2010). This is because in warmer climates appendages of endotherms that play a role in thermoregulation as a source of heat loss will be larger to allow for dissipation of heat loads (Greenberg, Cadena, Danner, & Tattersall, 2012). Using a butt‐ended ruler, a single person (RL) measured the flattened wing cord length from the carpal joint to the tip of the longest primary feather to an accuracy of 0.5 mm. We used the mean of two measurements in the analyses; repeatability (Lessells & Boag, 1987) for each species was ≥ 0.96.

### 2.3 Plumage color measurement

In each species, we measured plumage color for one yellow plumage patch (olive green in one species, see Table S1 for details). These colors are produced by yellow carotenoid pigments deposited within the feather structure, and can be reliably identified as carotenoid‐based by the unique shape of the reflectance spectrum (Delhey, 2015). The shape of the reflectance spectra of the color patches are very similar (average reflectance spectra are depicted in Fig. S2), indicative that they contain similar or identical yellow carotenoids.

**Figure 1:**
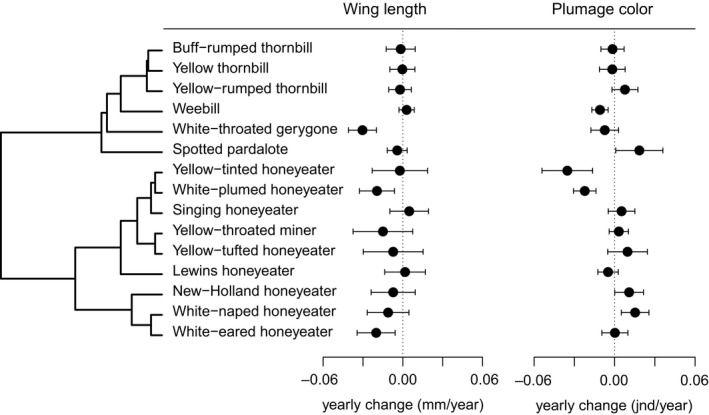
Estimates and 95% confidence intervals of temporal changes in wing length and carotenoid‐based plumage coloration for 15 species of Australian passerine birds. Estimates obtained from linear mixed models with wing length (mm) and plumage reflectance PC1 (jnd) as dependent variable, and decade of collection as predictor (see text for more details on mixed models). Phylogeny on the left represents patterns of relatedness between the species, for scientific names see Table S1

Generally, plumage color of museum specimens is an accurate representation of the color of live birds (Doucet & Hill, 2009). However, a certain degree of temporal change in color is expected due to fading and dirt accumulation, including in carotenoid‐based colors (McNett & Marchetti, 2005). particularly in older specimens (>50 years old, (Armenta, Dunn, & Whittingham, 2008)). The decrease seems to be more marked for total reflectance (brightness), which should not affect our measures of spectral shape (color), and for relative ultraviolet reflectance (Griggio, Hoi, & Pilastro, 2010; Griggio, Serra, & Pilastro, 2011; McNett & Marchetti, 2005), which is not a central component of carotenoid‐based colors. Nonetheless, we cannot exclude the possibility that color fading could contribute to a seeming temporal increase in plumage color intensity. However, if such effects are pervasive we should have seen overall positive temporal effects on color expression (recent specimens showing more intense color). This was not the case, as there was considerable heterogeneity in temporal effects, with some species showing increases and other declines in coloration (Figure 1), even though all colors are caused by the same mechanism (deposition of yellow carotenoids).

### 2.4 Reflectance spectra analysis

Reflectance measurements in the 300–700 nm range were obtained using a spectrometer (Avaspec 2048) connected to a xenon pulsed light source (Avalight‐Xe) through a bifurcated fiber optics cable and software Avasoft 6.2.1 (Avantes, Eerbeek, the Netherlands). Five reflectance spectra were taken per plumage patch, and spectra were processed using psychophysical models of avian color vision (Vorobyev, Osorio, Bennett, Marshall, & Cuthill, 1998). The five reflectance spectra collected per plumage patch were obtained from standardized location across the patch within each patch and species. Sampling across the patch (as opposed to only one spot) ensured that we captured spatial variation of color within the patch, effectively combining color intensity and patch size into one measurement. Visual modeling takes into account that birds see differently from humans, with different visual sensitivities and the ability to see into the ultraviolet range. The output from visual models are chromatic coordinates (*xyz*) that are derived from the relative stimulation of very short (VS), short (S), medium (M) and long (L) cones, and represent the coordinates of colors in the visual space of birds. For the visual models, we used cone sensitivities for violet‐sensitive (V‐type) eyes (Endler & Mielke, 2005), as this is the visual system reported for the Meliphagoidea (Ödeen, Håstad, & Alström, 2011). Cone proportions used corresponded to the average of the two Meliphagoidea species for which cone proportions are known (Blue‐faced honeyeater, *Entomyzon cyanotis* and Noisy miner, *Manorina melanocephala*, (Hart, 2001)). For all visual models, irradiance was set as standard daylight (d65, Vorobyev et al., 1998).

We obtained *xyz* coordinates using the r scripts provided in Delhey, Delhey, Kempenaers, and Peters (2015) which implement formulas from Cassey et al. (2008). We averaged the five visual coordinates obtained for each patch to derive a single set of chromatic coordinates for each specimen. We summarized chromatic variation into a single variable with a principal component (PC) analysis carried out on the *xyz* chromatic coordinates of each plumage patch using a covariance matrix to maintain original units (Delhey et al., 2015). These are called just noticeable differences (jnds) and represent chromatic differences in perceptual units (Vorobyev et al., 1998). The first principal component (PC1) explained 70%–96% (mean 91%) of chromatic variance for all species providing a single measure of color variation to be used in the analyses. In all cases higher values of PC1 correspond to more intensely colored carotenoid‐based plumage. PC1 correlates positively with carotenoid chroma, an index of yellow carotenoid deposition in the integument (Andersson & Prager, 2006), in all species (median Pearson correlation coefficient, *r* = .95, range = 0.9–0.99). Details of PC analysis are shown in Table S2.

### 2.5 Statistical analysis

The analysis of the data was done in two steps. First, we estimated temporal change in body size (wing length) and plumage color for each species while controlling for potentially confounding effects of other variables. Second we used these species‐specific estimates of temporal change to assess whether changes in plumage color are positively correlated with changes in wing length across the 15 species in our sample. We chose this approach, rather than testing the effect of plumage color on wing length directly, because we are mainly interested in assessing whether patterns of temporal change in these two phenotypic traits are consistent and correlated across species.

#### 2.5.1 Step 1. Quantifying temporal changes in body size and color within species

Linear mixed effect models with Gaussian error distributions were used to characterize temporal patterns in size and color separately for each species. For the analysis of change over time in body size, wing length was fitted as the response variable and decade of collection as a predictor variable, controlling for sex, altitude, and a residual autocovariate (RAC), as a substitute for latitude and longitude, to deal with spatial autocorrelation (Crase, Liedloff, & Wintle, 2012). Subspecies was also fitted as a random effect to account for differences in size or color (Delhey, Smith, & Peters, 2013). Decade of collection was used instead of year as this removed temporal autocorrelation in model residuals. For the analysis of temporal change in plumage reflectance, PC1 was fitted as the response variable with decade of collection as the predictor variable, again controlling for sex, subspecies, altitude and RAC as indicated above. In addition, season and season^2^ were included to account for change in plumage reflectance after molt resulting from abrasion and accumulation of dirt (Delhey, Burger, Fiedler, & Peters, 2010). Seasons were defined using 2‐month periods (e.g., January and February are defined as season one) and reflect the time since molt.

Using RAC reduced residual spatial autocorrelation in all species, as determined by Moran's I plots. No temporal autocorrelation was present in model residuals. Heterogeneity of variance was detected in some species (wing length models: Singing honeyeater*,* Lewin's honeyeater, white‐eared honeyeater; color models: White‐plumed honeyeater, Yellow‐throated Miner, white‐naped honeyeater, white‐eared honeyeater) after inspection of residual plots. In these cases we modeled the variance structure as outlined by Zuur, Ieno, Walker, Saveliev, & Smith (2009). Modeling the variance structure significantly improved models, as determined by AIC values while also reducing or eliminating heterogeneity of variance in residual plots.

The analysis above assumes that temporal changes are linear, which may not be true in all species (Gardner, Amano, Backwell, et al., 2014). To assess to what extent nonlinear relationships could be affecting patterns of temporal variation in wing length and color, we used generalized additive mixed models (GAMMs) with Gaussian error distributions. For the analysis of change over time in body size, wing length was fitted as the response variable and the year of collection (with a smooth function) as a predictor variable, controlling for sex, altitude and a RAC with subspecies as a random effect. For the analysis of temporal change in plumage reflectance, PC1 was fitted as the response variable with the year of collection (with a smooth function) as a predictor variable, again controlling for sex, subspecies (as a random effect), altitude and RAC. Season and season^2^ were also included as indicated above.

#### 2.5.2 Step 2. Across species analyses

We used Bayesian phylogenetic mixed models (Hadfield & Nakagawa, 2010) within a meta‐analytic framework (Nakagawa & Santos, 2012) to determine (1) whether there were consistent patterns of temporal changes in wing size and plumage color across species, and (2) whether temporal changes in color predicted temporal changes in size. In the first, we fitted two intercept‐only models with the species‐specific slope estimates from the GLMMs for temporal changes in wing length and color, respectively, as dependent variable (Nakagawa & Cuthill, 2007). In the second, we fitted a model with temporal change in wing length as dependent variable and temporal change in color as independent variable. Given that all temporal effect sizes within each trait have the same units, they do not need to be standardized and their standard errors were used to compute the measurement variance (Koricheva, Gurevitch, & Mengersen, 2013). In all analyses, we controlled for phylogenetic relatedness, including it as a random effect. Phylogenetic uncertainty was incorporated into the analyses by sampling phylogenies from of a posterior distribution of phylogenetic trees from Jetz, Thomas, Joy, Hartmann, and Mooers (2012) following Ross, Gardner, Hardy, and West (2013). We ran the model for 1,000 iterations for each phylogeny and saved the last Markov‐chain Monte Carlo sample. We used as starting values for the next phylogeny the latent variable values and variance components from the last iteration of the previous phylogeny. This process was repeated across 1,300 trees, and the results from the first 300 trees were discarded as burnin in each case, resulting in a posterior sample of 1,000 (Rubolini, Liker, Garamszegi, Møller, & Saino, 2015). We incorporated sampling error variances (the squared *SE* of each temporal effect estimate) using the “mev” argument in MCMCglmm (Hadfield & Nakagawa, 2010). Flat noninformative priors with low degree of belief were used for fixed effects, inverse gamma priors for residual variance and parameter expanded priors for phylogenetic effects (Hadfield, 2010, 2016). Model convergence was assessed by examining trace and autocorrelation plots. Heterogeneity (*I* ^2^), an indicator of how inconsistent patterns between species are, ranges from 0 to 1 (higher values indicate less consistency in patterns across species) and was computed following (Nakagawa & Santos, 2012). These analyses were repeated excluding those species with strong indication of nonlinear patterns of temporal changes as indicated by effective degrees of freedom exceeding 3 as shown by the smoothed term year in GAMMs (as in Gardner, Amano, Backwell, et al., 2014).

All statistical analyses were conducted in r version 2.15.2 (R Core Team, 2012). Linear mixed effect models and Bayesian phylogenetic mixed models were conducted using the packages “nlme” (Pinheiro, Bates, DebRoy, & Sarkar, 2011) and “MCMCglmm” (Hadfield, 2010) respectively. GAMMs were carried out using the packages “mgcv” (Wood, 2006). Phylogenetic tree and forest plots were created with the packages “ape” (Paradis, Claude, & Strimmer, 2004) and “metafor” (Viechtbauer, 2010), respectively.

## 3 Results

### 3.1 Temporal changes in body size

A significant decrease in wing length over time was observed for three of the 15 study species (Table 1, Figure 1, full results of linear models in Table S2 and scatterplots depicting temporal variation in Fig. S3). The white‐eared honeyeater*,* white‐plumed honeyeater*,* and white‐throated gerygone showed temporal decreases in wing length of 0.019–0.030 mm per year (Table 1). Nonsignificant trends of decreasing size were also recorded for the Spotted Pardalote and the white‐naped honeyeater (Table 1). Only three species showed increases in size over time, but these trends were not significant (Table 1). GAMMs analysis showed that temporal patterns of wing length change were largely linear except for the white‐throated gerygone (Table 1, full results in Table S3). Phylogenetic meta‐analysis indicated that, overall there was a trend of decreasing size, but this effect was not significant (effect = −0.007 mm/year, 95% CI = −0.019 to 0.004, *p* = .19). Variation in effect sizes across species was high (heterogeneity *I* ^2^, posterior mean = 0.90, 95% CI = 0.82–0.96). Phylogenetic signal, the proportion of the variation explained by phylogenetic relatedness (equivalent to Pagel's lambda, Hadfield & Nakagawa, 2010) in patterns of temporal change in wing length was low (posterior mean = 0.12, 95% CI = 1.79 × 10^−6^–0.447). Repeating the analysis excluding the white‐throated gerygone revealed similar results (−0.0005 mm/year, 95% CI = 0.017–0.008, *p* = .36).

### 3.2 Temporal changes in carotenoid‐based color

Significant temporal trends in plumage coloration were found for six of the 15 study species (Table 1, Figure 1, full results of linear models in Table S2; scatterplots depicting temporal variation in Fig. S4). The white‐plumed honeyeater, Yellow‐tinted honeyeater, and Weebill showed a significant decrease in color intensity over the study period, while the white‐naped honeyeater, New Holland honeyeater, and Spotted Pardalote showed a significant increase in color intensity (Table 1). Similar to changes in wing length, only the white‐throated gerygone showed strong nonlinear temporal patterns of change in the GAMMs analyses (Table 1, Table S3). Phylogenetic meta‐analysis indicated that there was no consistent pattern of color change over time across species (−0.0002 jnd/year, 95% CI = −0.014 to 0.012, *p* = .95), although there was very high heterogeneity (posterior mean = 0.94, 95% CI = 0.89 to 0.98). Phylogenetic signal was low and estimates highly variable (posterior mean = 0.14, 95% CI = 1.8 × 10^−7^–0.459). Excluding the white‐throated gerygone yielded similar results (−0.007 jnd/year, 95% CI = −0.014 to 0.015, *p* = .92).

### 3.3 Covariation between temporal trends in size and color

**Figure 2:**
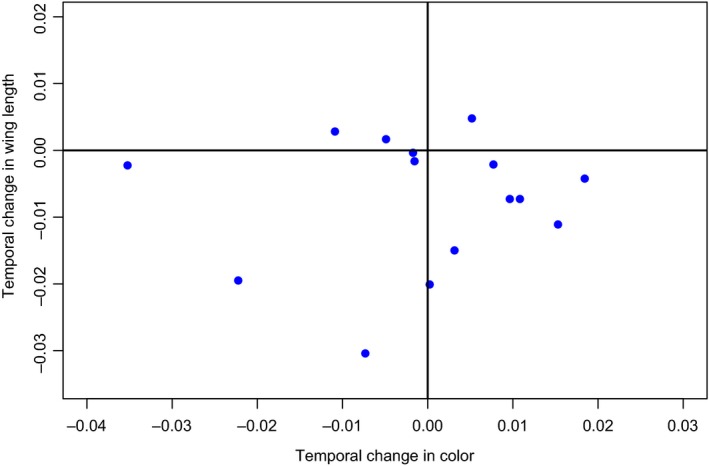
Temporal changes in size (mm/year) and carotenoid‐based plumage color (jnd/year) are not significantly correlated across 15 species of Australian Passerine birds

No significant overall correlation was found between size change and color change over time (slope = 0.071, 95% CI = −0.73 to 0.81, *p* = .84, Figure 2) while controlling for phylogenetic effects (posterior mean = 0.13, 95% CI = 3.7 × 10^−8^–0.46). The conclusions remain unchanged if we repeat the analyses excluding the white‐throated gerygone, the only species with strong nonlinear patterns of temporal variation (slope = 0.012, 95% CI = −0.750.88, *p* = .98). Only the white‐plumed honeyeater showed significant and concordant temporal changes in wing length and color (Table 1). In this case body size and carotenoid‐based plumage color intensity decreased significantly over the study period.

## 4 Discussion

In this study, we assessed the possibility that temporal changes in body size could be driven by changes in food availability by comparing temporal tends in size with temporal trends in a condition‐dependent trait: carotenoid‐based coloration. Our results clearly indicate that temporal changes in these two phenotypic traits are not significantly congruent. This suggests that changing food availability, in and of itself, is unlikely to be a driver of body size trends. Below we put these results in the context of other studies of body size change and discuss potential alternative explanations.

### 4.1 Temporal changes in carotenoid‐based color

Our study showed considerable heterogeneity in temporal changes in plumage coloration with some species showing increases and other declines in coloration (Figure 1), even though all colors are caused by deposition of yellow carotenoids. Our argument that such color is an indicator of past food availability in birds is based on a large body of evidence that food intake determines carotenoid‐based plumage color. Because animals are unable to synthesize carotenoids, all carotenoids present in the body need to be ingested, and producing intense carotenoid‐based coloration will require abundant, high‐quality food (for extensive reviews see Hill, 2006; McGraw, 2006). This is further exacerbated by other physiological costs associated with intake and processing of carotenoids in the body (for reviews see McGraw, 2006; Svensson and Wong, 2011). Indeed, experimental and observational studies, in captivity and in the wild, show that carotenoid‐based plumage color depends on carotenoid content of the diet (e.g., Isaksson & Andersson, 2007; McGraw & Hill, 2001; Peters, Roberts, Kurvers, & Delhey, 2012; Peters et al., 2008). Moreover, observational and experimental studies demonstrate that carotenoid‐based color varies with general body condition and quantity or quality of food intake in general (Hill, 2000; Jacot & Kempenaers, 2007; McGraw et al., 2005; Peters et al., 2006; Shawkey et al., 2006; Smith, Raberg, Ohlsson, Granbom, & Hasselquist, 2007). Additionally, a few studies found support for the three‐way link between environmental availability of abundant food of high quality, larger body size and more intense carotenoid‐based coloration (Eeva, Lehikoinen, & Ronka, 1998; Grether et al., 2001; Reudink et al., 2014).

Nonetheless, it is conceivable that the correlation between food quantity and quality and carotenoid‐based colors might weaken over longer timescales and during periods of environmental change. Carotenoid metabolism and transport are highly adaptable over evolutionary timescales, resulting in different pigment requirements between species (Craig & Foote, 2001; Svensson and Wong, 2011). Comparative evidence in birds suggests that the ability to acquire carotenoids evolves quickly in concert with the evolution of carotenoid‐based plumage colors (Simons, Maia, Leenknegt, & Verhulst, 2014). Such a compensatory change could obscure temporal changes in coloration (Grether, 2005). Second, factors other than diet may influence carotenoid color. For example, carotenoid color is affected by oxidative stress (Alonso‐Alvarez & Galvan, 2011), immune challenge (Fitze, Tschirren, Gasparini, & Richner, 2007; Peters, Delhey, Denk, & Kempenaers, 2004), parasites (Brawner, Hill, & Sundermann, 2000) cold stress (Eraud et al., 2007) and the duration of molt (Serra, Griggio, Licheri, & Pilastro, 2007). Temporal changes in such factors may either mask or enhance changes in plumage color directly in relation to diet. Such effects could be brought about for example by the appearance of new diseases (Zahn & Rothstein, 1999) or broad‐scale changes in physiological stress related to climate change (Kennedy, Lattin, Romero, & Dearborn, 2013; Treen, Hobson, Marchant, & Bortolotti, 2015). Third, changes in plumage color could be driven by pleiotropic effects of selection acting on other traits (Galeotti, Rubolini, Sacchi, & Fasola, 2009; Karell, Ahola, Karstinen, Valkama, & Brommer, 2011; Roulin, 2014). Thus, although carotenoid‐based color may indeed constitute a window on historical nutrition in birds, the fact that it is subject to caveats means that the lack of evidence that a broad‐scale change in carotenoid coloration is associated with size changes cannot be interpreted as conclusive evidence that changes in food availability do not contribute to body size changes. Nonetheless, given the available evidence that carotenoid‐based coloration generally is a reliable indicator of dietary intake and nutritional status, we suggest that it appears unlikely that broad‐based changes in nutrition have been the main driver of body size changes over the last 100 years in these bird species.

### 4.2 Temporal changes in body size

Most species in our sample show temporal decreases in body size (wing length), although there was substantial amount of heterogeneity in species responses (Figure 1). In general, decreases in size are considered consistent with global warming (but see Salewski et al., 2014), with smaller individuals coping better with the increasingly warmer conditions because they are better able to dissipate heat (Scholander, 1956). However, not all species decrease in size over time (Table 1, Gardner, Amano, Backwell, et al., 2014; Salewski et al., 2014) and mechanisms other than improved heat dissipation may be at play. Changes in food availability have been invoked to explain this variation (Yom‐Tov & Geffen, 2011).

Our data do not support the idea that changes in body size are linked with climate‐driven changes in food availability/nutrition as inferred by the quantification of carotenoid‐based coloration. Although there was considerable variation in patterns of temporal changes in size change and color change (Figure 1), temporal trends for color and size were not correlated across species, providing no evidence that broad‐scale change in food availability is driving size changes. While there are limitations to this approach (see above), these results are consistent with the only other study to empirically test for a temporal change in nutrition in Australian birds. Gardner et al. (2009) found no relationship between temporal declines in the body size of eight Australian passerines and changing nutritional condition, inferred from ptilochronology (feather growth rates). Taken together these results provide independent evidence that climate‐driven change in food availability or quality is not an overarching driver of temporal changes in body size, at least in birds. Nevertheless, the ability to deal with increasing heat loads requires allocation of energy for cooling which in turn is dependent on energy uptake. Hence, explanations for changing body size that relate to the need for increased heat dissipation need to consider metabolic processes, to which food availability and or quality are integral.

Potential alternative explanations, in particular for increases in body size, include the selective pressures exerted by extreme climatic events (McKechnie & Wolf, 2010) and changes in migratory behavior (Salewski et al., 2014). Extreme climatic events include heat and cold waves and periods with extreme low or high rainfall (Bailey & Pol, 2016). In general larger animals can withstand these stressful conditions better because they have greater energy or water reserves (Brown & Brown, 1999; Clark, 2009; McKechnie & Wolf, 2010). Thus an increase in the frequency of extreme events may lead to a trend of increases in body size, the so‐called starvation risk or fasting endurance hypothesis (Goodman et al., 2012). For example, increases in body size over time for a population of white‐plumed honeyeaters have been linked to increases in the frequency of heat waves, possibly via size‐dependent mortality (Gardner, Amano, Mackey, et al., 2014; Gardner, Amano, Sutherland, Clayton, & Peters, 2016). To what extent different species differ in susceptibility to these extreme events is unclear, but existing data suggest substantial variability (McKechnie et al., 2012) which could help explain variation in temporal changes in body size between species.

### 4.3 Future prospects

Our results have several broad implications: (1) Similar to temporal changes in size our data indicates that continent‐wide temporal changes in carotenoid‐based coloration vary between species, making it unlikely that these changes are simply due to fading of old specimens with time. Rather, (2) these changes could represent species‐specific processes of environmental and/or evolutionary change. However, (3) the fact that temporal changes in color were not correlated with changes in body size suggests that common mechanisms are unlikely to be the cause of these changes. In particular, it is unlikely that historical variation in food availability directly underlies patterns of temporal changes in size. The main strength of our study design, the concomitant measurement of size and color on museum specimens collected at a continental scale, could be supplemented by inclusion of complementary indicators of physiological condition. For example, corticosterone analysis of feathers from museum specimens can be used to as an indicator of physiological stress the individual experienced during molt (c.f., Kennedy et al., 2013; Treen et al., 2015) and historical changes in diet composition could be documented using stable isotope analysis (e.g., Chamberlain et al., 2005). Finally, local responses to small‐scale environmental changes might also be important contributors to size changes (Ozgo & Schilthuizen, 2012). Hence, studies like ours should be complemented by detailed monitoring of phenotypic and environmental change at the local or regional scale (Gardner, Amano, Mackey, et al., 2014; Salewski et al., 2014). Preferably, such studies would be replicated across the continent, but unfortunately, long‐term studies in general, and of bird color variation in particular, are rare (but see Karell et al., 2011 for an example).

## Conflict of Interest

None declared.
