# Supplementary material for: Are long‐term widespread avian body size changes related to food availability? A test using contemporaneous changes in carotenoid‐based color
Source: Ecol Evol. 2017 Mar 31;7(9):3157–66. doi: 10.1002/ece3.2739 (PMC5415506; doi:10.1002/ece3.2739)

**Supporting information**

Table S1. Common and scientific names of the study species, Family, sample sizes, colour patch measured and average and SE of wing length

Table S2. Results from linear mixed models

Table S3. Results from generalised additive mixed models.

Figure S1. Locations at which specimens were collected for each of the 15 study species measured. Points represent a single specimen and the size of each point indicates the body size of the individual, with larger points indicating larger size. Collection locations covered most of the species range.

Figure S2. Reflectance spectra for the carotenoid-based plumage patches measured.

Figure S3. Scatterplots depicting temporal changes in wing length

Figure S4. Scatterplots depicting temporal changes in carotenoid-based plumage colour.

| Table S1 Study species listed with both scientific and common name, family, sample size (n), colour patch measured and average wing length of the specimens measured (in mm). | | | | | | |
| --- | --- | --- | --- | --- | --- | --- |
| Scientific name | Common Name | Family | n | Colour patch measured | Wing (mm) | SE |
| *Acanthiza reguloides* | Buff-rumped Thornbill | Acanthizidae | 115 | Yellow underside | 52.9 | 0.19 |
| *Acanthiza nana* | Yellow Thornbill | Acanthizidae | 103 | Yellow underside | 49.6 | 0.18 |
| *Acanthiza chrysorrhoa* | Yellow-rumped Thornbill | Acanthizidae | 138 | Yellow rump | 58.4 | 0.27 |
| *Smicrornis brevirostris* | Weebill | Acanthizidae | 162 | Yellow underside | 49.9 | 0.15 |
| *Gerygone olivacea* | White-throated Gerygone | Acanthizidae | 82 | Yellow underside | 56.8 | 0.35 |
| *Pardalotus punctatus* | Spotted Pardalote | Pardalotidae | 143 | Yellow throat (males only) | 57.5 | 0.21 |
| *Ptilotula flavescens* | Yellow-tinted Honeyeater | Meliphagidae | 65 | Yellow face | 72.5 | 0.43 |
| *Ptilotula penicillatus* | White-plumed Honeyeater | Meliphagidae | 140 | Yellow cheek | 80.3 | 0.31 |
| *Gavicalis virescens* | Singing Honeyeater | Meliphagidae | 118 | Yellow cheek | 88.6 | 0.45 |
| *Manorina flavigula* | Yellow-throated Miner | Meliphagidae | 137 | Yellow wing edges | 125.3 | 0.52 |
| *Lichenostomus melanops* | Yellow-tufted Honeyeater | Meliphagidae | 130 | Yellow throat | 86.5 | 0.38 |
| *Meliphaga lewinii* | Lewin’s Honeyeater | Meliphagidae | 98 | Yellow cheek | 99.2 | 0.45 |
| *Phylidonyris novaehollandiae* | New Holland Honeyeater | Meliphagidae | 143 | Yellow wing patch | 76.7 | 0.30 |
| *Melithreptus lunatus* | White-naped Honeyeater | Meliphagidae | 109 | Olive-green back | 75.8 | 0.34 |
| *Nesoptilotis leucotis* | White-eared Honeyeater | Meliphagidae | 121 | Yellow underside | 91.2 | 0.58 |

**Table S2.** Linear mixed model (LMM) results analysing variation in wing length and carotenoid-based plumage coloration of 15 Australian passerine birds. RAC = residual autocovariate, as a substitute for latitude and longitude, to deal with spatial autocorrelation. See text for further details.

|  |  |  |  |  |  |  |  |  |  |  |  |  |  |  |
| --- | --- | --- | --- | --- | --- | --- | --- | --- | --- | --- | --- | --- | --- | --- |
| common name | scientific name |  | LMM - Wing length | | |  |  |  |  | LMM - Carotenoid-based colour | | | | |
|  |  |  | estimate | SE | df | t | p |  |  | estimate | SE | df | t | p |
| Buff-rumped Thornbill | *Acanthiza reguloides* | intercept | 51.6077 | 0.3639 | 107 | 141.83 | 0.000 |  | intercept | 0.5300 | 0.7012 | 103 | 0.76 | 0.451 |
|  |  | decade | -0.0016 | 0.0056 | 107 | -0.29 | 0.771 |  | decade | -0.0015 | 0.0044 | 103 | -0.35 | 0.726 |
|  |  | altitude | 0.0014 | 0.0007 | 107 | 2.08 | 0.040 |  | altitude | -0.0001 | 0.0005 | 103 | -0.19 | 0.849 |
|  |  | sex | 1.5780 | 0.3127 | 107 | 5.05 | 0.000 |  | sex | 0.3403 | 0.2475 | 103 | 1.37 | 0.172 |
|  |  | RAC | 0.1485 | 0.0511 | 107 | 2.90 | 0.004 |  | RAC | 0.2479 | 0.0580 | 103 | 4.27 | 0.000 |
|  |  |  |  |  |  |  |  |  | season | -0.2210 | 0.0944 | 103 | -2.34 | 0.021 |
|  |  |  |  |  |  |  |  |  | season^2 | -0.1317 | 0.0649 | 103 | -2.03 | 0.045 |
|  |  |  |  |  |  |  |  |  |  |  |  |  |  |  |
|  |  |  | estimate | SE | df | t | p |  |  | estimate | SE | df | t | p |
| Lewin's Honeyeater | *Meliphaga lewinii* | intercept | 94.3418 | 1.9248 | 92 | 49.01 | 0.000 |  | intercept | 0.4495 | 0.3367 | 88 | 1.34 | 0.185 |
|  |  | decade | 0.0017 | 0.0078 | 92 | 0.21 | 0.831 |  | decade | -0.0049 | 0.0039 | 88 | -1.26 | 0.211 |
|  |  | altitude | 0.0019 | 0.0007 | 92 | 2.86 | 0.005 |  | altitude | 0.0002 | 0.0003 | 88 | 0.64 | 0.522 |
|  |  | sex | 7.5054 | 0.4740 | 92 | 15.84 | 0.000 |  | sex | -0.4951 | 0.2686 | 88 | -1.84 | 0.069 |
|  |  | RAC | 0.3190 | 0.0912 | 92 | 3.50 | 0.001 |  | RAC | 0.1647 | 0.1024 | 88 | 1.61 | 0.111 |
|  |  |  |  |  |  |  |  |  | season | -0.1373 | 0.0833 | 88 | -1.65 | 0.103 |
|  |  |  |  |  |  |  |  |  | season^2 | -0.0942 | 0.0554 | 88 | -1.70 | 0.092 |
|  |  |  |  |  |  |  |  |  |  |  |  |  |  |  |
|  |  |  | estimate | SE | df | t | p |  |  | estimate | SE | df | t | p |
| New Holland Honeyeater | *Phylidonyris novaehollandiae* | intercept | 74.8784 | 0.8417 | 134 | 88.96 | 0.000 |  | intercept | 0.6496 | 0.5595 | 127 | 1.16 | 0.248 |
|  |  | decade | -0.0073 | 0.0085 | 134 | -0.86 | 0.392 |  | decade | 0.0108 | 0.0055 | 127 | 1.98 | 0.050 |
|  |  | altitude | -0.0015 | 0.0014 | 134 | -1.07 | 0.288 |  | altitude | -0.0001 | 0.0009 | 127 | -0.08 | 0.937 |
|  |  | sex | 3.7883 | 0.4940 | 134 | 7.67 | 0.000 |  | sex | -0.4754 | 0.3067 | 127 | -1.55 | 0.124 |
|  |  | RAC | 0.2116 | 0.0682 | 134 | 3.10 | 0.002 |  | RAC | 0.1620 | 0.0805 | 127 | 2.01 | 0.046 |
|  |  |  |  |  |  |  |  |  | season | -0.0143 | 0.1009 | 127 | -0.14 | 0.887 |
|  |  |  |  |  |  |  |  |  | season^2 | -0.0757 | 0.0658 | 127 | -1.15 | 0.253 |

|  |  |  |  |  |  |  |  |  |  |  |  |  |  |  |
| --- | --- | --- | --- | --- | --- | --- | --- | --- | --- | --- | --- | --- | --- | --- |
|  |  |  | estimate | SE | df | t | p |  |  | estimate | SE | df | t | p |
| Singing Honeyeater | *Gavicalis virescens* | intercept | 84.3055 | 1.3232 | 110 | 63.72 | 0.000 |  | intercept | -0.1421 | 0.3037 | 103 | -0.47 | 0.641 |
|  |  | decade | 0.0048 | 0.0074 | 110 | 0.64 | 0.521 |  | decade | 0.0052 | 0.0051 | 103 | 1.02 | 0.308 |
|  |  | altitude | -0.0002 | 0.0015 | 110 | -0.16 | 0.871 |  | altitude | -0.0016 | 0.0010 | 103 | -1.67 | 0.098 |
|  |  | sex | 6.3878 | 0.4928 | 110 | 12.96 | 0.000 |  | sex | 1.0649 | 0.3390 | 103 | 3.14 | 0.002 |
|  |  | RAC | 0.2947 | 0.0271 | 110 | 10.88 | 0.000 |  | RAC | 0.3185 | 0.0336 | 103 | 9.48 | 0.000 |
|  |  |  |  |  |  |  |  |  | season | -0.0807 | 0.1312 | 103 | -0.62 | 0.540 |
|  |  |  |  |  |  |  |  |  | season^2 | -0.1466 | 0.0861 | 103 | -1.70 | 0.092 |
|  |  |  |  |  |  |  |  |  |  |  |  |  |  |  |
|  |  |  | estimate | SE | df | t | p |  |  | estimate | SE | df | t | p |
| Spotted Pardalote | *Pardalotus punctatus* | intercept | 56.9854 | 1.0537 | 137 | 54.08 | 0.000 |  | intercept | 0.9173 | 0.3760 | 130 | 2.44 | 0.016 |
|  |  | decade | -0.0042 | 0.0038 | 137 | -1.11 | 0.270 |  | decade | 0.0184 | 0.0090 | 130 | 2.06 | 0.042 |
|  |  | altitude | -0.0005 | 0.0005 | 137 | -1.00 | 0.318 |  | altitude | -0.0006 | 0.0009 | 130 | -0.69 | 0.492 |
|  |  | RAC | 0.3110 | 0.0534 | 137 | 5.83 | 0.000 |  | RAC | 0.2740 | 0.0480 | 130 | 5.71 | 0.000 |
|  |  |  |  |  |  |  |  |  | season | -0.5955 | 0.2239 | 130 | -2.66 | 0.009 |
|  |  |  |  |  |  |  |  |  | season^2 | -0.1217 | 0.1578 | 130 | -0.77 | 0.442 |
|  |  |  |  |  |  |  |  |  |  |  |  |  |  |  |
|  |  |  | estimate | SE | df | t | p |  |  | estimate | SE | df | t | p |
| Weebill | *Smicrornis brevirostris* | intercept | 49.3044 | 0.5607 | 153 | 87.93 | 0.000 |  | intercept | -0.1491 | 0.5378 | 145 | -0.28 | 0.782 |
|  |  | decade | 0.0028 | 0.0029 | 153 | 0.97 | 0.336 |  | decade | -0.0109 | 0.0031 | 145 | -3.52 | 0.001 |
|  |  | altitude | 0.0008 | 0.0005 | 153 | 1.64 | 0.104 |  | altitude | 0.0000 | 0.0005 | 145 | -0.04 | 0.971 |
|  |  | sex | 0.8825 | 0.2042 | 153 | 4.32 | 0.000 |  | sex | -0.3514 | 0.2048 | 145 | -1.72 | 0.088 |
|  |  | RAC | 0.2407 | 0.0300 | 153 | 8.02 | 0.000 |  | RAC | 0.3142 | 0.0289 | 145 | 10.87 | 0.000 |
|  |  |  |  |  |  |  |  |  | season | -0.1386 | 0.0850 | 145 | -1.63 | 0.105 |
|  |  |  |  |  |  |  |  |  | season^2 | 0.0801 | 0.0518 | 145 | 1.55 | 0.125 |

|  |  |  |  |  |  |  |  |  |  |  |  |  |  |  |
| --- | --- | --- | --- | --- | --- | --- | --- | --- | --- | --- | --- | --- | --- | --- |
|  |  |  | estimate | SE | df | t | p |  |  | estimate | SE | df | t | p |
| White-eared Honeyeater | *Nesoptilotis leucotis* | intercept | 83.3391 | 0.3197 | 113 | 260.64 | 0.000 |  | intercept | -1.1566 | 0.4119 | 108 | -2.81 | 0.006 |
|  |  | decade | -0.0201 | 0.0073 | 113 | -2.75 | 0.007 |  | decade | 0.0002 | 0.0049 | 108 | 0.05 | 0.962 |
|  |  | altitude | -0.0001 | 0.0008 | 113 | -0.15 | 0.880 |  | altitude | 0.0000 | 0.0004 | 108 | 0.04 | 0.966 |
|  |  | sex | 10.4742 | 0.4836 | 113 | 21.66 | 0.000 |  | sex | 0.7810 | 0.2940 | 108 | 2.66 | 0.009 |
|  |  | RAC | 0.3466 | 0.0388 | 113 | 8.93 | 0.000 |  | RAC | 0.2587 | 0.0674 | 108 | 3.84 | 0.000 |
|  |  |  |  |  |  |  |  |  | season | -0.2470 | 0.1034 | 108 | -2.39 | 0.019 |
|  |  |  |  |  |  |  |  |  | season^2 | -0.1564 | 0.0745 | 108 | -2.10 | 0.038 |
|  |  |  |  |  |  |  |  |  |  |  |  |  |  |  |
|  |  |  | estimate | SE | df | t | p |  |  | estimate | SE | df | t | p |
| White-naped Honeyeater | *Melithreptus lunatus* | intercept | 73.8097 | 1.7459 | 103 | 42.28 | 0.000 |  | intercept | -0.1050 | 0.4852 | 98 | -0.22 | 0.829 |
|  |  | decade | -0.0111 | 0.0080 | 103 | -1.39 | 0.169 |  | decade | 0.0153 | 0.0053 | 98 | 2.91 | 0.004 |
|  |  | altitude | 0.0013 | 0.0008 | 103 | 1.61 | 0.111 |  | altitude | 0.0002 | 0.0005 | 98 | 0.47 | 0.638 |
|  |  | sex | 4.4190 | 0.5725 | 103 | 7.72 | 0.000 |  | sex | 0.4965 | 0.3659 | 98 | 1.36 | 0.178 |
|  |  | RAC | 0.3146 | 0.0540 | 103 | 5.83 | 0.000 |  | RAC | 0.1551 | 0.0669 | 98 | 2.32 | 0.022 |
|  |  |  |  |  |  |  |  |  | season | -0.1742 | 0.1255 | 98 | -1.39 | 0.168 |
|  |  |  |  |  |  |  |  |  | season^2 | -0.1455 | 0.0918 | 98 | -1.58 | 0.116 |
|  |  |  |  |  |  |  |  |  |  |  |  |  |  |  |
|  |  |  | estimate | SE | df | t | p |  |  | estimate | SE | df | t | p |
| White-plumed Honeyeater | *Ptilotula penicillatus* | intercept | 77.4565 | 1.1474 | 133 | 67.51 | 0.000 |  | intercept | 1.1026 | 1.0736 | 130 | 1.03 | 0.306 |
|  |  | decade | -0.0195 | 0.0067 | 133 | -2.89 | 0.004 |  | decade | -0.0222 | 0.0043 | 130 | -5.18 | 0.000 |
|  |  | altitude | -0.0009 | 0.0009 | 133 | -0.95 | 0.342 |  | altitude | 0.0001 | 0.0006 | 130 | 0.20 | 0.843 |
|  |  | sex | 4.3970 | 0.3865 | 133 | 11.38 | 0.000 |  | sex | -0.2154 | 0.2485 | 130 | -0.87 | 0.388 |
|  |  | RAC | 0.2650 | 0.0340 | 133 | 7.79 | 0.000 |  | RAC | 0.2075 | 0.0360 | 130 | 5.76 | 0.000 |
|  |  |  |  |  |  |  |  |  | season | 0.1645 | 0.0971 | 130 | 1.69 | 0.093 |
|  |  |  |  |  |  |  |  |  | season^2 | -0.1673 | 0.0613 | 130 | -2.73 | 0.007 |

|  |  |  |  |  |  |  |  |  |  |  |  |  |  |  |
| --- | --- | --- | --- | --- | --- | --- | --- | --- | --- | --- | --- | --- | --- | --- |
|  |  |  | estimate | SE | df | t | p |  |  | estimate | SE | df | t | p |
| White-throated Gerygone | *Gerygone olivacea* | intercept | 54.5457 | 1.1150 | 75 | 48.92 | 0.000 |  | intercept | -0.0571 | 0.4017 | 71 | -0.14 | 0.887 |
|  |  | decade | -0.0304 | 0.0054 | 75 | -5.67 | 0.000 |  | decade | -0.0073 | 0.0053 | 71 | -1.39 | 0.168 |
|  |  | altitude | 0.0017 | 0.0008 | 75 | 2.15 | 0.035 |  | altitude | -0.0005 | 0.0007 | 71 | -0.75 | 0.456 |
|  |  | sex | 1.9613 | 0.4209 | 75 | 4.66 | 0.000 |  | sex | 0.1567 | 0.3962 | 71 | 0.40 | 0.694 |
|  |  | RAC | 0.2862 | 0.0446 | 75 | 6.41 | 0.000 |  | RAC | 0.3467 | 0.0649 | 71 | 5.34 | 0.000 |
|  |  |  |  |  |  |  |  |  | season | -0.3667 | 0.1945 | 71 | -1.88 | 0.064 |
|  |  |  |  |  |  |  |  |  | season^2 | -0.0751 | 0.0978 | 71 | -0.77 | 0.445 |
|  |  |  |  |  |  |  |  |  |  |  |  |  |  |  |
|  |  |  | estimate | SE | df | t | p |  |  | estimate | SE | df | t | p |
| Yellow-rumped Thornbill | *Acanthiza chrysorrhoa* | intercept | 57.3136 | 0.2934 | 130 | 195.32 | 0.000 |  | intercept | -0.4755 | 0.4445 | 126 | -1.07 | 0.287 |
|  |  | decade | -0.0021 | 0.0043 | 130 | -0.49 | 0.626 |  | decade | 0.0077 | 0.0049 | 126 | 1.57 | 0.119 |
|  |  | altitude | 0.0006 | 0.0006 | 130 | 1.00 | 0.319 |  | altitude | 0.0010 | 0.0007 | 126 | 1.50 | 0.136 |
|  |  | sex | 1.2927 | 0.2864 | 130 | 4.51 | 0.000 |  | sex | 0.7285 | 0.3282 | 126 | 2.22 | 0.028 |
|  |  | RAC | 0.2785 | 0.0453 | 130 | 6.14 | 0.000 |  | RAC | 0.3025 | 0.0402 | 126 | 7.53 | 0.000 |
|  |  |  |  |  |  |  |  |  | season | -0.1776 | 0.1242 | 126 | -1.43 | 0.155 |
|  |  |  |  |  |  |  |  |  | season^2 | -0.1814 | 0.0786 | 126 | -2.31 | 0.023 |
|  |  |  |  |  |  |  |  |  |  |  |  |  |  |  |
|  |  |  | estimate | SE | df | t | p |  |  | estimate | SE | df | t | p |
| Yellow-throated Miner | *Manorina flavigula* | intercept | 122.9042 | 1.9437 | 130 | 63.23 | 0.000 |  | intercept | 0.1252 | 0.2312 | 128 | 0.54 | 0.589 |
|  |  | decade | -0.0150 | 0.0115 | 130 | -1.31 | 0.193 |  | decade | 0.0031 | 0.0037 | 128 | 0.86 | 0.392 |
|  |  | altitude | -0.0058 | 0.0021 | 130 | -2.82 | 0.006 |  | altitude | 0.0002 | 0.0007 | 128 | 0.24 | 0.810 |
|  |  | sex | 6.0944 | 0.7114 | 130 | 8.57 | 0.000 |  | sex | 0.0120 | 0.2406 | 128 | 0.05 | 0.960 |
|  |  | RAC | 0.2656 | 0.0261 | 130 | 10.19 | 0.000 |  | RAC | 0.2978 | 0.0467 | 128 | 6.38 | 0.000 |
|  |  |  |  |  |  |  |  |  | season | 0.1812 | 0.1059 | 128 | 1.71 | 0.089 |
|  |  |  |  |  |  |  |  |  | season^2 | -0.1864 | 0.0793 | 128 | -2.35 | 0.020 |

|  |  |  |  |  |  |  |  |  |  |  |  |  |  |  |
| --- | --- | --- | --- | --- | --- | --- | --- | --- | --- | --- | --- | --- | --- | --- |
|  |  |  | estimate | SE | df | t | p |  |  | estimate | SE | df | t | p |
| Yellow-tinted Honeyeater | *Ptilotula flavescens* | intercept | 70.8967 | 1.3534 | 59 | 52.38 | 0.000 |  | intercept | 0.2797 | 0.9846 | 57 | 0.28 | 0.777 |
|  |  | decade | -0.0023 | 0.0106 | 59 | -0.21 | 0.833 |  | decade | -0.0352 | 0.0096 | 57 | -3.66 | 0.001 |
|  |  | altitude | 0.0046 | 0.0025 | 59 | 1.83 | 0.073 |  | altitude | -0.0055 | 0.0021 | 57 | -2.69 | 0.009 |
|  |  | sex | 5.3966 | 0.5650 | 59 | 9.55 | 0.000 |  | sex | 0.3322 | 0.4942 | 57 | 0.67 | 0.504 |
|  |  | RAC | 0.1789 | 0.0613 | 59 | 2.92 | 0.005 |  | RAC | 0.0618 | 0.0652 | 57 | 0.95 | 0.347 |
|  |  |  |  |  |  |  |  |  | season | -0.5082 | 0.2300 | 57 | -2.21 | 0.031 |
|  |  |  |  |  |  |  |  |  | season^2 | -0.0900 | 0.1578 | 57 | -0.57 | 0.571 |
|  |  |  |  |  |  |  |  |  |  |  |  |  |  |  |
|  |  |  | estimate | SE | df | t | p |  |  | estimate | SE | df | t | p |
| Yellow-tufted Honeyeater | *Lichenostomus melanops* | intercept | 85.1153 | 1.7987 | 124 | 47.32 | 0.000 |  | intercept | 0.7378 | 0.6812 | 121 | 1.08 | 0.281 |
|  |  | decade | -0.0073 | 0.0115 | 124 | -0.63 | 0.527 |  | decade | 0.0096 | 0.0075 | 121 | 1.28 | 0.204 |
|  |  | altitude | 0.0020 | 0.0014 | 124 | 1.43 | 0.154 |  | altitude | 0.0015 | 0.0009 | 121 | 1.70 | 0.093 |
|  |  | sex | 3.3527 | 0.6228 | 124 | 5.38 | 0.000 |  | sex | 0.0196 | 0.4098 | 121 | 0.05 | 0.962 |
|  |  | RAC | 0.2122 | 0.0347 | 124 | 6.11 | 0.000 |  | RAC | 0.2022 | 0.0926 | 121 | 2.18 | 0.031 |
|  |  |  |  |  |  |  |  |  | season | 0.1498 | 0.1365 | 121 | 1.10 | 0.275 |
|  |  |  |  |  |  |  |  |  | season^2 | -0.2796 | 0.0903 | 121 | -3.10 | 0.002 |
|  |  |  |  |  |  |  |  |  |  |  |  |  |  |  |
|  |  |  | estimate | SE | df | t | p |  |  | estimate | SE | df | t | p |
| Yellow Thornbill | *Acanthiza nana* | intercept | 49.6173 | 0.9360 | 96 | 53.01 | 0.000 |  | intercept | 2.3932 | 1.4719 | 94 | 1.63 | 0.107 |
|  |  | decade | -0.0004 | 0.0048 | 96 | -0.08 | 0.940 |  | decade | -0.0017 | 0.0049 | 94 | -0.35 | 0.725 |
|  |  | altitude | 0.0008 | 0.0008 | 96 | 0.95 | 0.347 |  | altitude | 0.0009 | 0.0008 | 94 | 1.12 | 0.267 |
|  |  | sex | 1.1256 | 0.2784 | 96 | 4.04 | 0.000 |  | sex | -0.2326 | 0.2799 | 94 | -0.83 | 0.408 |
|  |  | RAC | 0.2843 | 0.0733 | 96 | 3.88 | 0.000 |  | RAC | 0.4013 | 0.0551 | 94 | 7.28 | 0.000 |
|  |  |  |  |  |  |  |  |  | season | -0.3880 | 0.1055 | 94 | -3.68 | 0.000 |
|  |  |  |  |  |  |  |  |  | season^2 | -0.1031 | 0.0592 | 94 | -1.74 | 0.085 |

**Table S3.** Generalised additive mixed model (GAMM) results analysing variation in wing length and carotenoid-based plumage coloration of 15 Australian passerine birds. RAC = residual autocovariate, as a substitute for latitude and longitude, to deal with spatial autocorrelation. See text for further details.

| common name | scientific name | GAMM - Wing length | |  |  |  |  | GAMM - Carotenoid-based plumage coloration (PC1) | | | | |
| --- | --- | --- | --- | --- | --- | --- | --- | --- | --- | --- | --- | --- |
|  |  |  |  |  |  |  |  |  |  |  |  |  |
| Buff-rumped thornbill | *Acanthiza reguloides* |  | estimate | SE | t | p |  |  | estimate | SE | t | p |
|  |  | intercept | 51.8174 | 0.2490 | 208.061 | 0.0000 |  | intercept | 0.4847 | 0.2213 | 2.1897 | 0.0308 |
|  |  | sex | 1.5684 | 0.3094 | 5.070 | 0.0000 |  | season | -0.1876 | 0.0907 | -2.0677 | 0.0411 |
|  |  | altitude | 0.0011 | 0.0006 | 1.780 | 0.0778 |  | season^2 | -0.1972 | 0.0643 | -3.0659 | 0.0028 |
|  |  | RAC | 0.1638 | 0.0467 | 3.506 | 0.0007 |  | sex | 0.3179 | 0.2380 | 1.3356 | 0.1846 |
|  |  |  | edf | Ref.df | F | p |  | altitude | 0.0003 | 0.0005 | 0.7106 | 0.4789 |
|  |  | s(year) | 1.0000 | 1.0000 | 0.029 | 0.8643 |  | RAC | 0.3001 | 0.0529 | 5.6775 | 0.0000 |
|  |  |  |  |  |  |  |  |  | edf | Ref.df | F | p |
|  |  |  |  |  |  |  |  | s(year) | 2.8627 | 2.8627 | 5.6378 | 0.0016 |
|  |  |  |  |  |  |  |  |  |  |  |  |  |
| Lewin's honeyeater | *Meliphaga lewinii* |  | estimate | SE | t | p |  |  | estimate | SE | t | p |
|  |  | intercept | 94.1087 | 0.4185 | 224.882 | 0.0000 |  | intercept | 0.5529 | 0.2761 | 2.0027 | 0.0483 |
|  |  | sex | 7.6121 | 0.5029 | 15.136 | 0.0000 |  | season | -0.1490 | 0.0823 | -1.8112 | 0.0735 |
|  |  | altitude | 0.0016 | 0.0007 | 2.505 | 0.0140 |  | season^2 | -0.0975 | 0.0552 | -1.7657 | 0.0809 |
|  |  | RAC | 0.3016 | 0.0408 | 7.396 | 0.0000 |  | sex | -0.5204 | 0.2669 | -1.9499 | 0.0543 |
|  |  |  | edf | Ref.df | F | p |  | altitude | 0.0002 | 0.0003 | 0.4892 | 0.6259 |
|  |  | s(year) | 1.0000 | 1.0000 | 0.164 | 0.6864 |  | RAC | 0.1699 | 0.1017 | 1.6715 | 0.0981 |
|  |  |  |  |  |  |  |  |  | edf | Ref.df | F | p |
|  |  |  |  |  |  |  |  | s(year) | 1.0000 | 1.0000 | 1.8357 | 0.1788 |
|  |  |  |  |  |  |  |  |  |  |  |  |  |
| New Holland honeyeater | *Phylidonyris novaehollandiae* |  | estimate | SE | t | p |  |  | estimate | SE | t | p |
|  |  | intercept | 74.6861 | 0.4057 | 184.072 | 0.0000 |  | intercept | 0.6077 | 0.2914 | 2.0853 | 0.0390 |
|  |  | sex | 3.8104 | 0.4977 | 7.656 | 0.0000 |  | season | 0.0158 | 0.0961 | 0.1644 | 0.8697 |
|  |  | altitude | -0.0019 | 0.0014 | -1.386 | 0.1681 |  | season^2 | -0.0624 | 0.0640 | -0.9741 | 0.3318 |
|  |  | RAC | 0.2597 | 0.0565 | 4.595 | 0.0000 |  | sex | -0.4890 | 0.3042 | -1.6073 | 0.1104 |
|  |  |  | edf | Ref.df | F | p |  | altitude | -0.0001 | 0.0008 | -0.1204 | 0.9043 |
|  |  | s(year) | 1.0000 | 1.0000 | 0.456 | 0.5008 |  | RAC | 0.2443 | 0.0468 | 5.2218 | 0.0000 |
|  |  |  |  |  |  |  |  |  | edf | Ref.df | F | p |
|  |  |  |  |  |  |  |  | s(year) | 1.0000 | 1.0000 | 5.8760 | 0.0167 |
|  |  |  |  |  |  |  |  |  |  |  |  |  |
| Singing honeyeater | *Gavicalis virescens* |  | estimate | SE | t | p |  |  | estimate | SE | t | p |
|  |  | intercept | 84.5342 | 0.3872 | 218.312 | 0.0000 |  | intercept | -0.3028 | 0.2965 | -1.0210 | 0.3096 |
|  |  | sex | 6.2944 | 0.5033 | 12.507 | 0.0000 |  | season | -0.0541 | 0.1283 | -0.4218 | 0.6741 |
|  |  | altitude | -0.0011 | 0.0015 | -0.708 | 0.4802 |  | season^2 | -0.1126 | 0.0838 | -1.3436 | 0.1819 |
|  |  | RAC | 0.2903 | 0.0229 | 12.696 | 0.0000 |  | sex | 1.1027 | 0.3331 | 3.3106 | 0.0013 |
|  |  |  | edf | Ref.df | F | p |  | altitude | -0.0017 | 0.0010 | -1.7865 | 0.0769 |
|  |  | s(year) | 1.8059 | 1.8059 | 1.731 | 0.1796 |  | RAC | 0.3087 | 0.0314 | 9.8170 | 0.0000 |
|  |  |  |  |  |  |  |  |  | edf | Ref.df | F | p |
|  |  |  |  |  |  |  |  | s(year) | 1.0000 | 1.0000 | 1.2734 | 0.2617 |
|  |  |  |  |  |  |  |  |  |  |  |  |  |
| Spotted pardalote | *Pardalotus punctatus* |  | estimate | SE | t | p |  |  | estimate | SE | t | p |
|  |  | intercept | 57.0293 | 0.1273 | 447.879 | 0.0000 |  | intercept | 0.9329 | 0.3767 | 2.4768 | 0.0145 |
|  |  | altitude | -0.0004 | 0.0004 | -0.962 | 0.3379 |  | season | -0.5777 | 0.2239 | -2.5803 | 0.0110 |
|  |  | RAC | 0.3355 | 0.0375 | 8.947 | 0.0000 |  | season^2 | -0.1359 | 0.1579 | -0.8606 | 0.3910 |
|  |  |  | edf | Ref.df | F | p |  | sex | -0.0008 | 0.0009 | -0.8804 | 0.3802 |
|  |  | s(year) | 1 | 1.0000 | 2.131 | 0.1465 |  | altitude | 0.2645 | 0.0463 | 5.7092 | 0.0000 |
|  |  |  |  |  |  |  |  | RAC | edf | Ref.df | F | p |
|  |  |  |  |  |  |  |  | s(year) | 1.0000 | 1.0000 | 3.6308 | 0.0589 |
|  |  |  |  |  |  |  |  |  |  |  |  |  |
| Weebill | *Smicrornis brevirostris* |  | estimate | SE | t | p |  |  | estimate | SE | t | p |
|  |  | intercept | 49.3474 | 0.1820 | 271.145 | 0.0000 |  | intercept | -0.0518 | 0.1640 | -0.3157 | 0.7527 |
|  |  | sex | 0.8342 | 0.1979 | 4.215 | 0.0000 |  | season | -0.1256 | 0.0802 | -1.5664 | 0.1194 |
|  |  | altitude | 0.0008 | 0.0004 | 1.937 | 0.0545 |  | season^2 | 0.0563 | 0.0489 | 1.1529 | 0.2508 |
|  |  | RAC | 0.2733 | 0.0227 | 12.031 | 0.0000 |  | sex | -0.3151 | 0.1872 | -1.6836 | 0.0944 |
|  |  |  | edf | Ref.df | F | p |  | altitude | -0.0002 | 0.0004 | -0.5915 | 0.5551 |
|  |  | s(year) | 1.0000 | 1.0000 | 0.878 | 0.3501 |  | RAC | 0.3127 | 0.0168 | 18.5952 | 0.0000 |
|  |  |  |  |  |  |  |  |  | edf | Ref.df | F | p |
|  |  |  |  |  |  |  |  | s(year) | 1.0000 | 1.0000 | 13.3622 | 0.0004 |
|  |  |  |  |  |  |  |  |  |  |  |  |  |

| White-eared honeyeater | *Nesoptilotis leucotis* |  | estimate | SE | t | p |  |  | estimate | SE | t | p |
| --- | --- | --- | --- | --- | --- | --- | --- | --- | --- | --- | --- | --- |
|  |  | intercept | 83.2679 | 0.5621 | 148.132 | 0.0000 |  | intercept | -1.2418 | 0.3888 | -3.1935 | 0.0018 |
|  |  | sex | 10.4098 | 0.6582 | 15.814 | 0.0000 |  | season | -0.0516 | 0.1112 | -0.4644 | 0.6433 |
|  |  | altitude | -0.0003 | 0.0009 | -0.389 | 0.6977 |  | season^2 | -0.0888 | 0.0784 | -1.1336 | 0.2594 |
|  |  | RAC | 0.3363 | 0.0441 | 7.631 | 0.0000 |  | sex | 0.5947 | 0.3207 | 1.8541 | 0.0664 |
|  |  |  | edf | Ref.df | F | p |  | altitude | -0.0004 | 0.0004 | -0.9941 | 0.3224 |
|  |  | s(year) | 1.0000 | 1.0000 | 5.724 | 0.0183 |  | RAC | 0.2475 | 0.0586 | 4.2242 | 0.0000 |
|  |  |  |  |  |  |  |  |  | edf | Ref.df | F | p |
|  |  |  |  |  |  |  |  | s(year) | 2.8896 | 2.8896 | 7.3961 | 0.0002 |
|  |  |  |  |  |  |  |  |  |  |  |  |  |
| White-naped honeyeater | *Melithreptus lunatus* |  | estimate | SE | t | p |  |  | estimate | SE | t | p |
|  |  | intercept | 74.1913 | 0.5149 | 144.090 | 0.0000 |  | intercept | -0.1702 | 0.1234 | -1.3786 | 0.1711 |
|  |  | sex | 4.3275 | 0.5709 | 7.580 | 0.0000 |  | season | -0.1471 | 0.0936 | -1.5711 | 0.1193 |
|  |  | altitude | 0.0010 | 0.0008 | 1.237 | 0.2190 |  | season^2 | 0.4926 | 0.3715 | 1.3261 | 0.1879 |
|  |  | RAC | 0.3016 | 0.0420 | 7.176 | 0.0000 |  | sex | 0.0001 | 0.0005 | 0.1065 | 0.9154 |
|  |  |  | edf | Ref.df | F | p |  | altitude | 0.1678 | 0.0665 | 2.5243 | 0.0132 |
|  |  | s(year) | **2.2214** | 2.2214 | 4.267 | 0.0140 |  | RAC | edf | Ref.df | F | p |
|  |  |  |  |  |  |  |  | s(year) | 1.0000 | 1.0000 | 9.1185 | 0.0032 |
|  |  |  |  |  |  |  |  |  |  |  |  |  |
| White-plumed honeyeater | *Ptilotula penicillatus* |  | estimate | SE | t | p |  |  | estimate | SE | t | p |
|  |  | intercept | 77.5644 | 0.2789 | 278.120 | 0.0000 |  | intercept | 1.0989 | 0.3697 | 2.9724 | 0.0035 |
|  |  | sex | 4.4308 | 0.3789 | 11.695 | 0.0000 |  | season | 0.1425 | 0.0974 | 1.4632 | 0.1458 |
|  |  | altitude | -0.0006 | 0.0009 | -0.710 | 0.4788 |  | season^2 | -0.1634 | 0.0617 | -2.6471 | 0.0091 |
|  |  | RAC | 0.2761 | 0.0265 | 10.436 | 0.0000 |  | sex | -0.2364 | 0.2456 | -0.9623 | 0.3377 |
|  |  |  | edf | Ref.df | F | p |  | altitude | 0.0001 | 0.0006 | 0.2246 | 0.8226 |
|  |  | s(year) | **1.6895** | 1.6895 | 5.113 | 0.0116 |  | RAC | 0.2279 | 0.0304 | 7.5084 | 0.0000 |
|  |  |  |  |  |  |  |  |  | edf | Ref.df | F | p |
|  |  |  |  |  |  |  |  | s(year) | 1.5353 | 1.5353 | 26.0342 | 0.0000 |
|  |  |  |  |  |  |  |  |  |  |  |  |  |

| White-throated gerygone | *Gerygone olivacea* |  | estimate | SE | t | p |  |  | estimate | SE | t | p |
| --- | --- | --- | --- | --- | --- | --- | --- | --- | --- | --- | --- | --- |
|  |  | intercept | 54.7550 | 0.3298 | 166.035 | 0.0000 |  | intercept | -0.1902 | 0.3280 | -0.5799 | 0.5639 |
|  |  | sex | 1.7651 | 0.4008 | 4.404 | 0.0000 |  | season | -0.4818 | 0.1948 | -2.4732 | 0.0158 |
|  |  | altitude | 0.0026 | 0.0008 | 3.346 | 0.0013 |  | season^2 | -0.1765 | 0.0927 | -1.9045 | 0.0610 |
|  |  | RAC | 0.2719 | 0.0288 | 9.444 | 0.0000 |  | sex | 0.5336 | 0.3694 | 1.4445 | 0.1531 |
|  |  |  | edf | Ref.df | F | p |  | altitude | -0.0019 | 0.0007 | -2.7763 | 0.0071 |
|  |  | s(year) | **3.8158** | 3.8158 | 12.415 | 0.0000 |  | RAC | 0.2577 | 0.0590 | 4.3686 | 0.0000 |
|  |  |  |  |  |  |  |  |  | edf | Ref.df | F | p |
|  |  |  |  |  |  |  |  | s(year) | 4.4148 | 4.4148 | 7.9565 | 0.0000 |
|  |  |  |  |  |  |  |  |  |  |  |  |  |
| Yellow thornbill | *Acanthiza nana* |  | estimate | SE | t | p |  |  | estimate | SE | t | p |
|  |  | intercept | 49.5605 | 0.2352 | 210.714 | 0.0000 |  | intercept | 2.6257 | 0.2328 | 11.2808 | 0.0000 |
|  |  | sex | 1.1370 | 0.2731 | 4.163 | 0.0001 |  | season | -0.4179 | 0.0912 | -4.5841 | 0.0000 |
|  |  | altitude | 0.0019 | 0.0007 | 2.829 | 0.0057 |  | season^2 | -0.0990 | 0.0513 | -1.9308 | 0.0565 |
|  |  | RAC | 0.3384 | 0.0637 | 5.316 | 0.0000 |  | sex | -0.1877 | 0.2418 | -0.7762 | 0.4395 |
|  |  |  | edf | Ref.df | F | p |  | altitude | 0.0001 | 0.0006 | 0.2414 | 0.8098 |
|  |  | s(year) | 1.0000 | 1.0000 | 0.330 | 0.5667 |  | RAC | 0.3568 | 0.0195 | 18.3040 | 0.0000 |
|  |  |  |  |  |  |  |  |  | edf | Ref.df | F | p |
|  |  |  |  |  |  |  |  | s(year) | 1.0000 | 1.0000 | 0.0148 | 0.9034 |
|  |  |  |  |  |  |  |  |  |  |  |  |  |
| Yellow-tinted honeyeater | *Ptilotula flavescens* |  | estimate | SE | t | p |  |  | estimate | SE | t | p |
|  |  | intercept | 70.5241 | 0.5647 | 124.880 | 0.0000 |  | intercept | -0.2265 | 0.4642 | -0.4880 | 0.6274 |
|  |  | sex | 5.4102 | 0.5627 | 9.615 | 0.0000 |  | season | -0.4169 | 0.2193 | -1.9010 | 0.0623 |
|  |  | altitude | 0.0045 | 0.0025 | 1.804 | 0.0762 |  | season^2 | -0.0583 | 0.1523 | -0.3830 | 0.7031 |
|  |  | RAC | 0.2088 | 0.0575 | 3.633 | 0.0006 |  | sex | 0.3023 | 0.4909 | 0.6158 | 0.5404 |
|  |  |  | edf | Ref.df | F | p |  | altitude | -0.0055 | 0.0020 | -2.6915 | 0.0093 |
|  |  | s(year) | 1.0000 | 1.0000 | 0.014 | 0.9065 |  | RAC | 0.1040 | 0.0530 | 1.9629 | 0.0545 |
|  |  |  |  |  |  |  |  |  | edf | Ref.df | F | p |
|  |  |  |  |  |  |  |  | s(year) | 1.0000 | 1.0000 | 14.9676 | 0.0003 |
|  |  |  |  |  |  |  |  |  |  |  |  |  |

| Yellow-rumped thornbill | *Acanthiza chrysorrhoa* |  | estimate | SE | t | p |  |  | estimate | SE | t | p |
| --- | --- | --- | --- | --- | --- | --- | --- | --- | --- | --- | --- | --- |
|  |  | intercept | 57.3082 | 0.2229 | 257.108 | 0.0000 |  | intercept | -0.3708 | 0.3013 | -1.2308 | 0.2207 |
|  |  | sex | 1.2951 | 0.2807 | 4.614 | 0.0000 |  | season | -0.1599 | 0.1189 | -1.3449 | 0.1810 |
|  |  | altitude | 0.0006 | 0.0006 | 1.042 | 0.2995 |  | season^2 | -0.1795 | 0.0769 | -2.3354 | 0.0211 |
|  |  | RAC | 0.2836 | 0.0428 | 6.623 | 0.0000 |  | sex | 0.8048 | 0.3258 | 2.4702 | 0.0148 |
|  |  |  | edf | Ref.df | F | p |  | altitude | 0.0008 | 0.0007 | 1.1963 | 0.2338 |
|  |  | s(year) | 1 | 1.0000 | 0.332 | 0.5654 |  | RAC | 0.2791 | 0.0330 | 8.4463 | 0.0000 |
|  |  |  |  |  |  |  |  |  | edf | Ref.df | F | p |
|  |  |  |  |  |  |  |  | s(year) | 1.0000 | 1.0000 | 1.1818 | 0.2790 |
|  |  |  |  |  |  |  |  |  |  |  |  |  |
| Yellow-throated miner | *Manorina flavigula* |  | estimate | SE | t | p |  |  | estimate | SE | t | p |
|  |  | intercept | 121.8776 | 0.5782 | 210.792 | 0.0000 |  | intercept | 0.0966 | 0.2452 | 0.3938 | 0.6944 |
|  |  | sex | 6.1808 | 0.7154 | 8.640 | 0.0000 |  | season | 0.1186 | 0.1112 | 1.0661 | 0.2884 |
|  |  | altitude | -0.0071 | 0.0019 | -3.653 | 0.0004 |  | season^2 | -0.1556 | 0.0933 | -1.6690 | 0.0975 |
|  |  | RAC | 0.2765 | 0.0258 | 10.726 | 0.0000 |  | sex | -0.0254 | 0.2601 | -0.0976 | 0.9224 |
|  |  |  | edf | Ref.df | F | p |  | altitude | -0.0004 | 0.0007 | -0.6320 | 0.5285 |
|  |  | s(year) | 1.0000 | 1.0000 | 4.844 | 0.0294 |  | RAC | 0.2466 | 0.0497 | 4.9635 | 0.0000 |
|  |  |  |  |  |  |  |  |  | edf | Ref.df | F | p |
|  |  |  |  |  |  |  |  | s(year) | 1.0000 | 1.0000 | 0.1813 | 0.6710 |
|  |  |  |  |  |  |  |  |  |  |  |  |  |
| Yellow-tufted honeyeater | *Lichenostomus melanops* |  | estimate | SE | t | p |  |  | estimate | SE | t | p |
|  |  | intercept | 85.0003 | 0.8205 | 103.598 | 0.0000 |  | intercept | 0.5521 | 0.3555 | 1.5531 | 0.1230 |
|  |  | sex | 3.3296 | 0.6339 | 5.253 | 0.0000 |  | season | 0.1383 | 0.1378 | 1.0032 | 0.3177 |
|  |  | altitude | 0.0008 | 0.0014 | 0.539 | 0.5907 |  | season^2 | -0.3014 | 0.0917 | -3.2854 | 0.0013 |
|  |  | RAC | 0.2015 | 0.0340 | 5.925 | 0.0000 |  | sex | 0.0916 | 0.4116 | 0.2226 | 0.8242 |
|  |  |  | edf | Ref.df | F | p |  | altitude | 0.0010 | 0.0008 | 1.1266 | 0.2621 |
|  |  | s(year) | 1 | 1.0000 | 0.012 | 0.9127 |  | RAC | 0.1923 | 0.0937 | 2.0518 | 0.0424 |
|  |  |  |  |  |  |  |  |  | edf | Ref.df | F | p |
|  |  |  |  |  |  |  |  | s(year) | 1.9871 | 1.9871 | 3.2451 | 0.0427 |

**Figure S1.** Locations at which specimens were collected for each of the 15 study species measured. Points represent a single specimen and the size of each point indicates the body size of the individual, with larger points indicating larger size. Collection locations covered most of the species range.

**
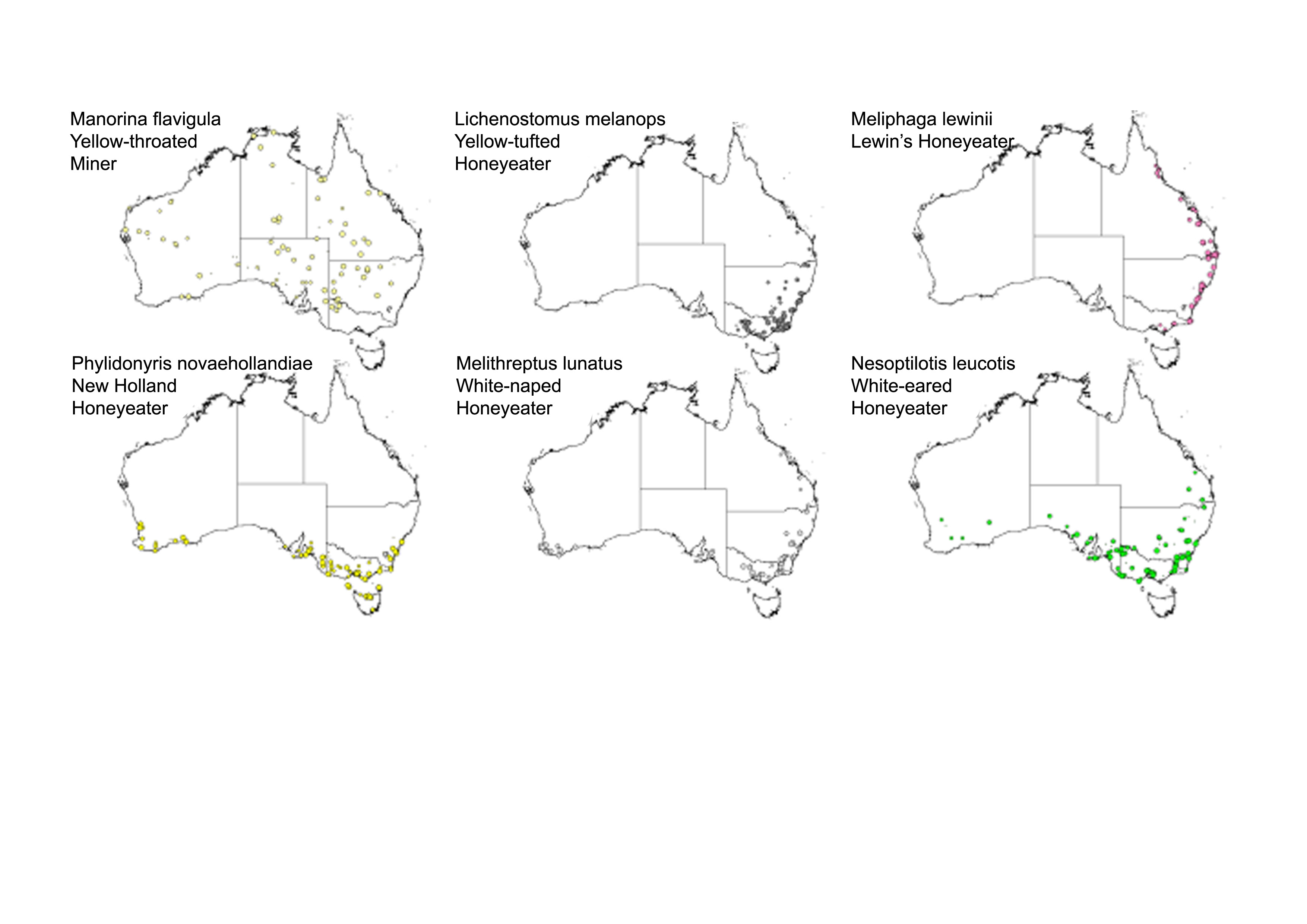
**

**
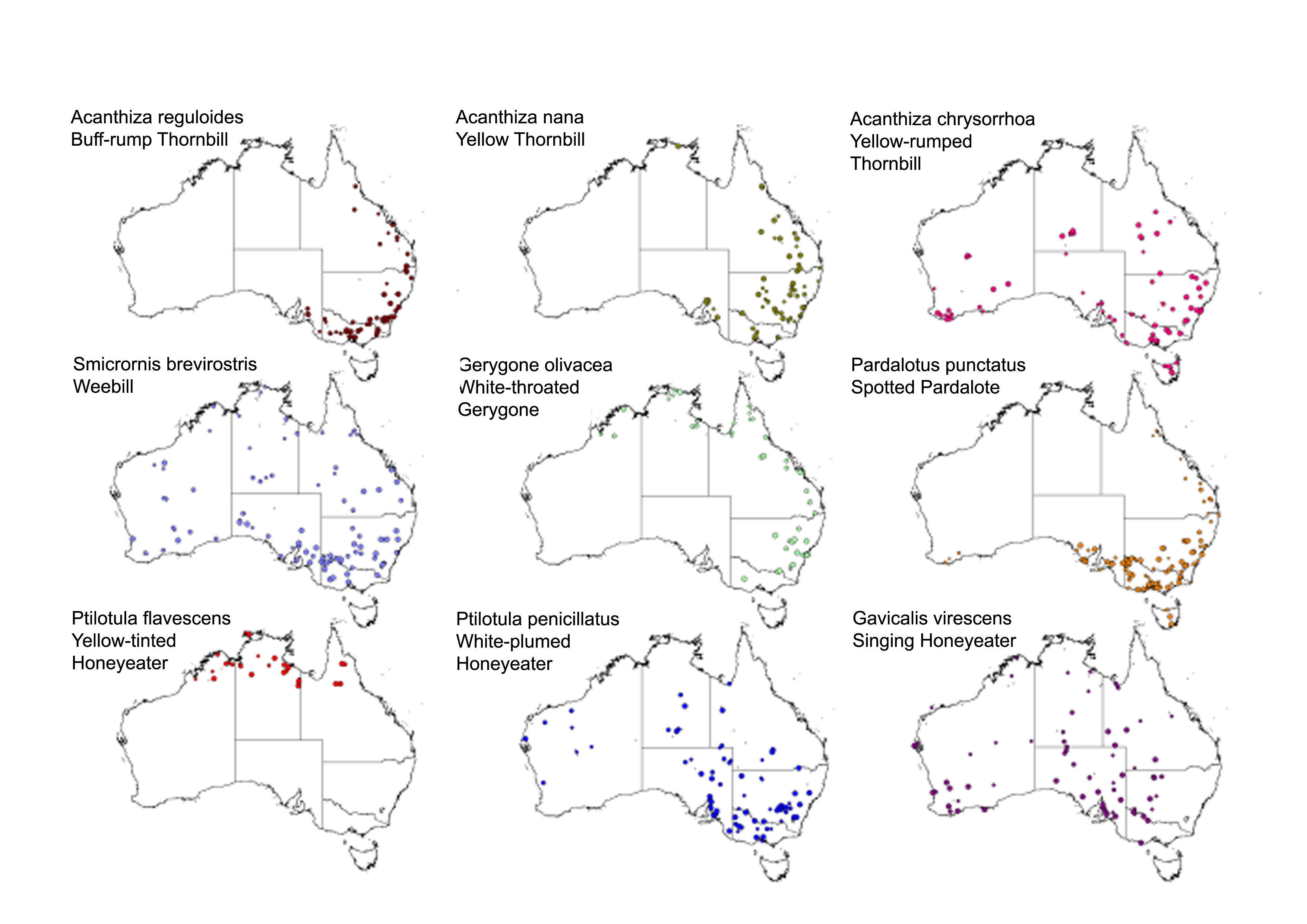
**

**Figure S2.** Reflectance spectra (300-700 nm) for the carotenoid-based plumage patches of 15 species of Australian passerine birds. Blue symbols represent the average spectra and grey ones the variation between individuals.


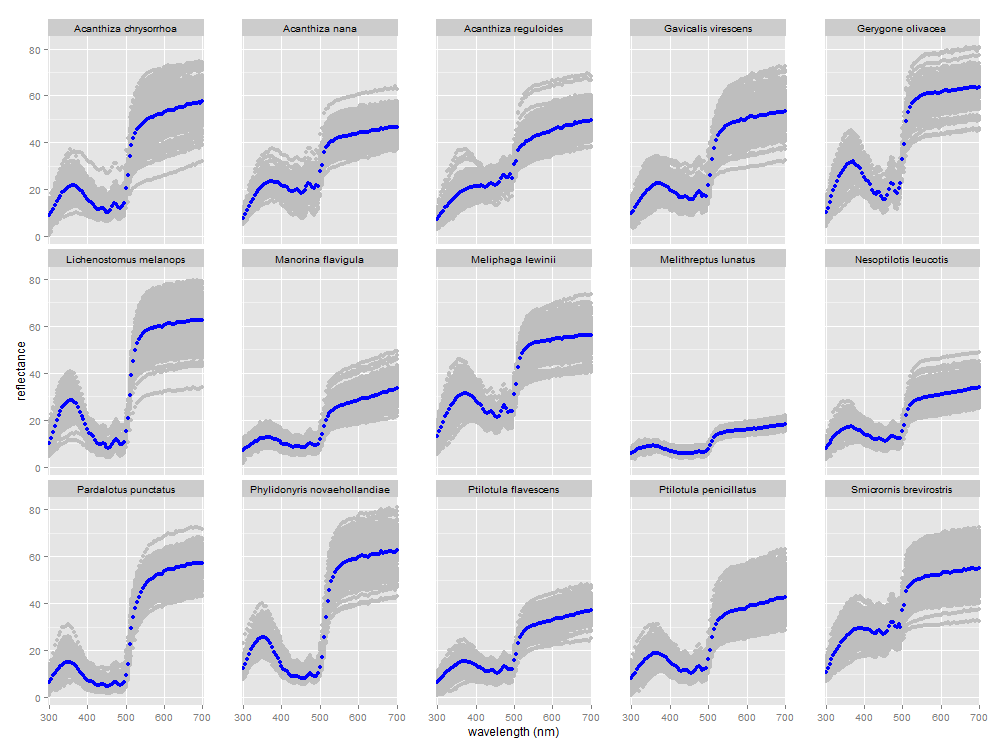


**Figure S3.** Scatterplots depicting temporal changes in wing length for 15 species of Australian passerines. Lines depict linear temporal trends as estimated by linear mixed models (see text for more details) for males and females.


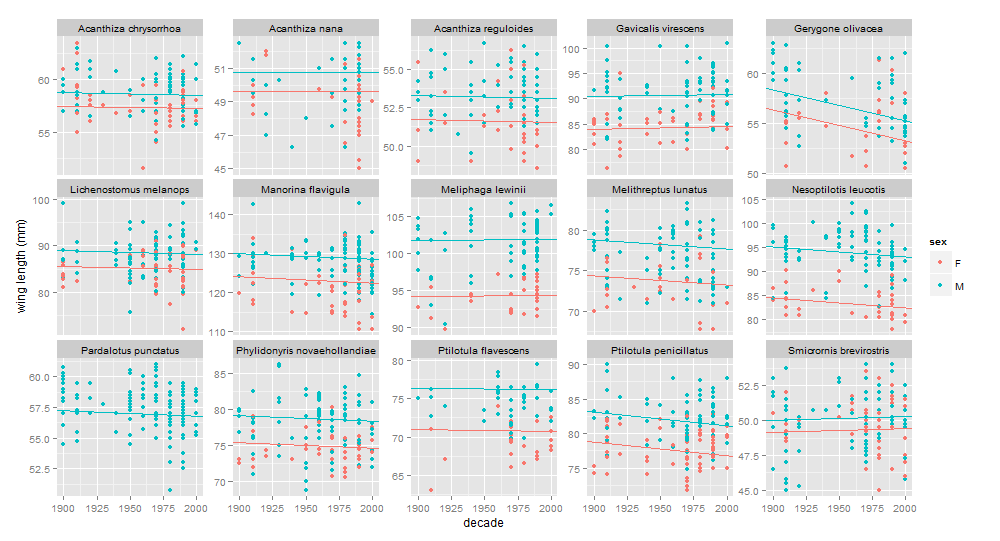


**Figure S4.** Scatterplots depicting temporal changes in carotenoid-based plumage colour for 15 species of Australian passerines. Lines depict linear temporal trends as estimated by linear mixed models (see text for more details) for males and females.


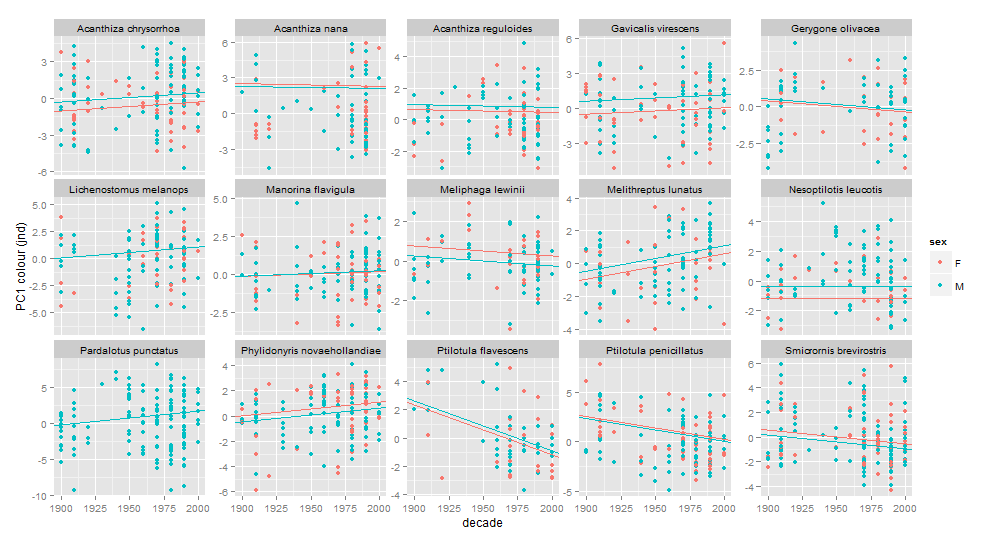

Supplement: Supplementary file 1 [file ECE3-7-3157-s001.docx]
